# Case report: A study on the pathological diagnosis of oral fibrosarcoma in a giant panda

**DOI:** 10.3389/fvets.2024.1500679

**Published:** 2025-02-04

**Authors:** Zongliang Xiong, Shanshan Ling, Caiwu Li, Linhua Deng, Ming He, Chengdong Wang, Desheng Li, Zhengli Chen

**Affiliations:** ^1^Experimental Animal Disease Model Research Laboratory, School of Veterinary Medicine, Sichuan Agricultural University, Chengdu, China; ^2^Key Laboratory of State Forestry and Grassland Administration on the Giant Panda, China Conservation and Research Center for the Giant Panda, Chengdu, China

**Keywords:** case report, giant panda, oral fibrosarcoma, pathology diagnostic research, biopsy

## Abstract

A female giant panda presented with a large, hard mass on the right cheek, which was investigated at the Dujiangyan Base of the China Conservation and Research Center for the Giant Panda with computed tomography (CT) scans and biopsy of the oral mass, as well as venous blood sampling. Hematological tests revealed elevated levels of the tumor marker CA724, along with infectious disease, anemia, hepatic damage, and renal failure. Imaging studies identified a soft tissue mass in the right cheek area. Macroscopic pathological changes, histopathological features, and immunohistochemical analysis were consistent with a low-grade malignant fibrosarcoma within the oral cavity, indicating malignant potential. This case represents the first report of an oral tumor in a giant panda, providing valuable data for future clinical diagnoses of neoplasms in this species.

## Introduction

The giant panda (*Ailuropoda melanoleuca*), the only mammal within the order Carnivora, family Ursidae, subfamily Ailuropodinae, and genus Ailuropoda, is an endangered and rare animal, affording it first-class state protection in China. It is referred to as a “living fossil” and “Chinese national treasure”, and research into its diseases, as well as prevention and control, have garnered significant attention. With the enhancement of animal protection measures, the life expectancy of giant pandas has increased, and the occurrence of tumors has risen correspondingly. Consequently, research on tumors in giant pandas has gained increasing significance. There are relatively few reports documenting giant panda tumor cases. At present, the documented cases include only types such as ovarian cancer, pancreatic ductal adenocarcinoma, seminoma, cutaneous hemangioma, and conjunctival angiosarcoma (1–5). This results in a scarcity of clinical diagnostic data pertaining to giant panda tumors, with a particular deficiency in detection data and reports from live sampling.

This report concentrates on a case of neoplasm in an aged giant panda identified during clinical feeding and management procedures. The preliminary diagnosis was informed by hematology, serum biochemistry, and oncologic blood markers. Subsequent diagnostic workups, including imaging and histopathological examination, facilitated further diagnosis of biopsied samples obtained from the living panda, resulting in a confirmed diagnosis of oral fibrosarcoma. Oral fibrosarcoma is a highly invasive malignant neoplasm, characterized by an insidious onset and limited initial lesion size. In veterinary oncology, oral fibrosarcoma is frequently diagnosed in dogs, cats, and various other species. Oral tumors and tumor-like lesions in dogs and cats are often categorized as proliferative, inflammatory, or benign conditions, typically affecting middle-aged animals, without gender predisposition. The most common lesion in dogs is gingival hyperplasia, followed by peripheral odontogenic fibroma, and in cats, the most prevalent condition is lymphoplasmacytic stomatitis. The oral tumor in this report of a giant panda is presumed to originate from oral bleeding induced by consuming bamboo sticks. Chronic irritation led to the growth of specific tumors within the oral cavity, ultimately progressing to low-grade malignant fibroma. Consequently, this case report establishes a reasonably standardized and systematic diagnostic approach for oral tumors in giant pandas, offering a tangible benchmark for early detection and diagnosis of such tumors in giant pandas moving forward.

## Case description

### Medical history investigation

Upon reviewing the medical records at the Dujiangyan Base of the China Conservation and Research Center for the Giant Panda, we obtained insights into the giant panda's clinical presentations and medical history. The detailed information is as follows: Ye Ye, a female giant panda, was born on September 25, 1999. At the age of 20, a physical examination detected a mass, measuring approximately 1 cm × 1 cm × 1 cm, beneath the mucosa of her right cheek. Subsequently, at age 21, she was transferred from the Shenshuping Base to the Dujiangyan Base, with no significant changes noted during this interval. Two years post-transfer, oral bleeding was observed, and examination identified a large, hard mass on her right cheek. Four months subsequent to this discovery, tissue from Ye Ye's oral mass and venous blood were collected for further diagnostic analysis.

### Clinical examination

**Figure 1:**
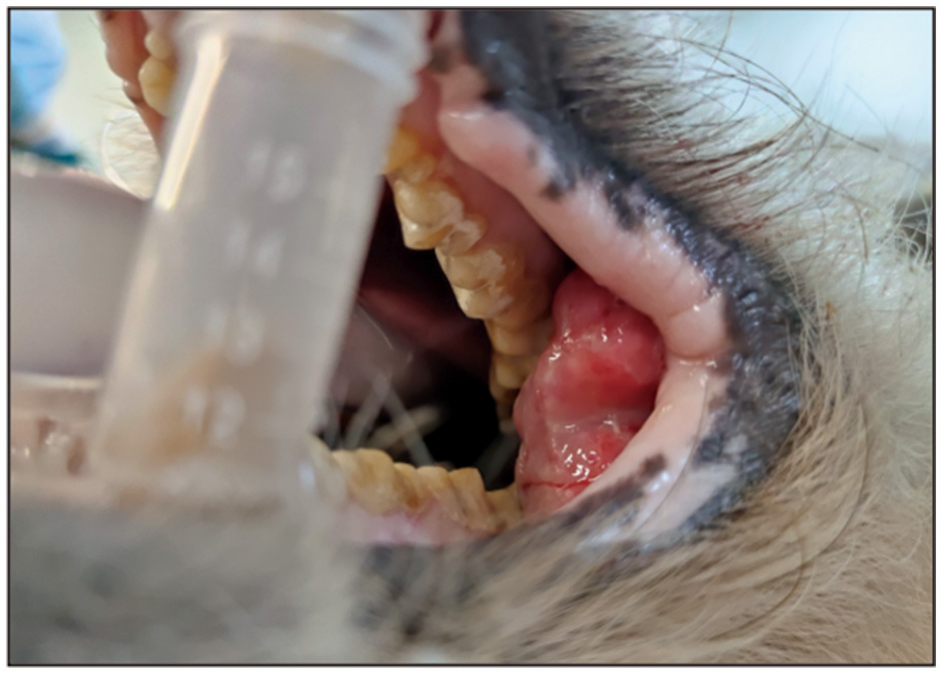
Oral growths in afflicted giant pandas.

Routine examinations, including live sample collection, were conducted: with a body temperature of 37.3°C, a respiratory rate of 20 breaths/min, and a heart rate of 80 beats/min. Clinical examination revealed that the lesion was located beneath the buccal mucosa within the oral cavity. There were no signs of excessive salivation, exophthalmos, facial edema or deformity, epistaxis, weight loss, halitosis, dysphagia, or oral pain, nor was there cervical lymph node enlargement, or loose teeth. A smooth-surfaced, firm, multi-lobed tumor was visualized inside the oral cavity (Figure 1).

## Results

### Hematology tests

Routine hematological analysis: the leukocyte count (WBC) is unremarkable, whereas neutrophil percentage (Neu%) and count (Neu) are elevated. Conversely, lymphocyte percentage (Lym%) and count (Lym#) are reduced, and eosinophil percentage (Eos%) and count (Eos) are likewise decreased. These findings suggest a potential infectious or neoplastic process in the giant panda Ye Ye, supported by indicators of lymphatic tissue compromise. The erythrocyte count (RBC) for Ye Ye is stable; however, decrements in hemoglobin (HGB), hematocrit (HCT), mean corpuscular volume (MCV), mean corpuscular hemoglobin (MCH), red cell distribution width standard deviation (RDW-SD), and mean platelet volume (MPV) are consistent with a diagnosis of nutritional anemia. Furthermore, a marked increase in the platelet count (PLT) suggests a possible link to malignancy (Table 1). Blood biochemical analysis reveals elevated activities of alanine aminotransferase (ALT) and aspartate aminotransferase (AST), with reduced albumin (ALB) levels and elevated globulin (GLO) levels. Additionally, total bilirubin (TBil) levels are diminished, γ-glutamyltransferase (GGT) levels are increased, and alkaline phosphatase (ALP) levels are elevated. These findings collectively suggest liver tissue pathology and hepatocellular damage in the giant panda Ye Ye. Elevated serum creatinine (CREA) and reduced uric acid (UA) levels indicate impaired renal function, specifically a decline in glomerular filtration rate, in the giant panda Ye Ye (Table 1). Coagulation profile reveals: Prothrombin time (PT) is prolonged, and fibrinogen concentration (Fbg) is reduced. Fibrinogen, identified as coagulation factor I, is a principal protein in the coagulation cascade. An elevated thrombin time (TT) suggests potential liver dysfunction, infection, or malignancy in the giant panda Ye Ye (Table 1). Serum tumor marker analysis: carbohydrate antigen 72-4 (CA724) levels were elevated, a marker for epithelial neoplasms. Hence, the presence of an epithelial tumor in the giant panda Ye Ye is suggested (Table 1).

**Table 1:** Hematology test of the giant panda called YeYe.

**Test type**	**Test Item**	**Results**	**Units of Measurement**	**Range**
Routine blood test	WBC	8.84	10e9/L	3.5–9.5
	Neu%	84.8	%	50–70
	Lym%	11.4	%	20–40
	Mon%	3.3	%	3.0–8
	Eos%	0.2	%	0.5–5.
	Bas%	0.3	%	0–1.0
	Neu#	7.5	10e9/L	2.00–7.00
	Lym#	1.01	10e9/L	1.1–3.2
	Mon#	0.29	10e9/L	0.10–0.60
	Eos#	0.01	10e9/L	0.02–0.52
	Bas#	0.03	10e9/L	0.00–0.10
	RBC	5.05	10e12/L	4.0–5.5
	HGB	86	g/L	120–160
	HCT	25.1	%	35.0–45.0
	MCV	49.8	fL	82.0–100.0
	MCH	17	pg	27.0–34.0
	MCHC	342	g/L	316–354
	RDW-CV	14.4	%	11.0–16.0
	RDW-SD	30.1	fL	39.0–53.9
	PLT	638	10e9/L	80–300
	MPV	5	fL	9.4–12.5
	PDW	14.4	fL	9.8–16.2
	PCT	0.319	%	0.16–0.38
Blood biochemistry test	ALT	175	U/L	5–40
	AST	122	U/L	8–40
	ALT/AST	0.7		0.8–2.0
	TP	62.9	g/L	60–85
	ALB	21.8	g/L	35–55
	GLO	41.1	g/L	20–40
	A/G	0.5		1.5–2.5
	TBil	1.69	umol/L	3.4–20.5
	DBil	1.61	umol/L	0–6.8
	IBi1	0.08	umol/L	0–15
	GGT	52	u/L	11–50
	ALP	386	u/L	40–150
	TBA	31.2	umol/L	0–10
	PA	7	mg/L	200–400
	CHE	580	u/L	4,000–11,000
	BUN	8.11	mmol/L	2.86–8.2
	CREA	112.06	umol/L	21.5–104
	UA	48	umol/L	208–428
	CYS-C	0.04	mg/L	0.51–1.09
	GLU	2.65	mmol/L	2.75–22
	TC	3.12	mmol/L	0–5.2
	TG	1.99	mmol/L	0–1.7
	HDL	1.95	mmol/L	0.83–1.96
	LDL	1.08	mmol/L	0–3.12
	ApoA1	0.01	g/L	1–1.6
	ApoB	0.02	g/L	0.6–1.10
	eGFR	1750.89	mL/min/1	
Coagulation test	PT	23.2	Sec	10–40
	APTT	32.5	Sec	20–40
	Fbg	1.84	g/L	45,326
	TT	24.7	Sec	14–21
	INR	2.09		0.8–1.25
Blood tumor marker detection	AFB	0.16	ng/mL	0–9
	CEA	0.06	ng/mL	0–5
	CA153	0	U/mL	0–14
	CA19-9	< 0.8	U/mL	0–25
	CA125	1.1	U/mL	0–35
	CA724	30.19	U/mL	0–6.9

The values marked in blue are below the reference range, and those marked in red above it.

Given the findings from the hematological tests, it is tentatively concluded that the giant panda Ye Ye may have a neoplastic condition and may additionally be experiencing infectious diseases, anemia, hepatic impairment, renal dysfunction, among other conditions.

### Imaging assessment

**Figure 2:**
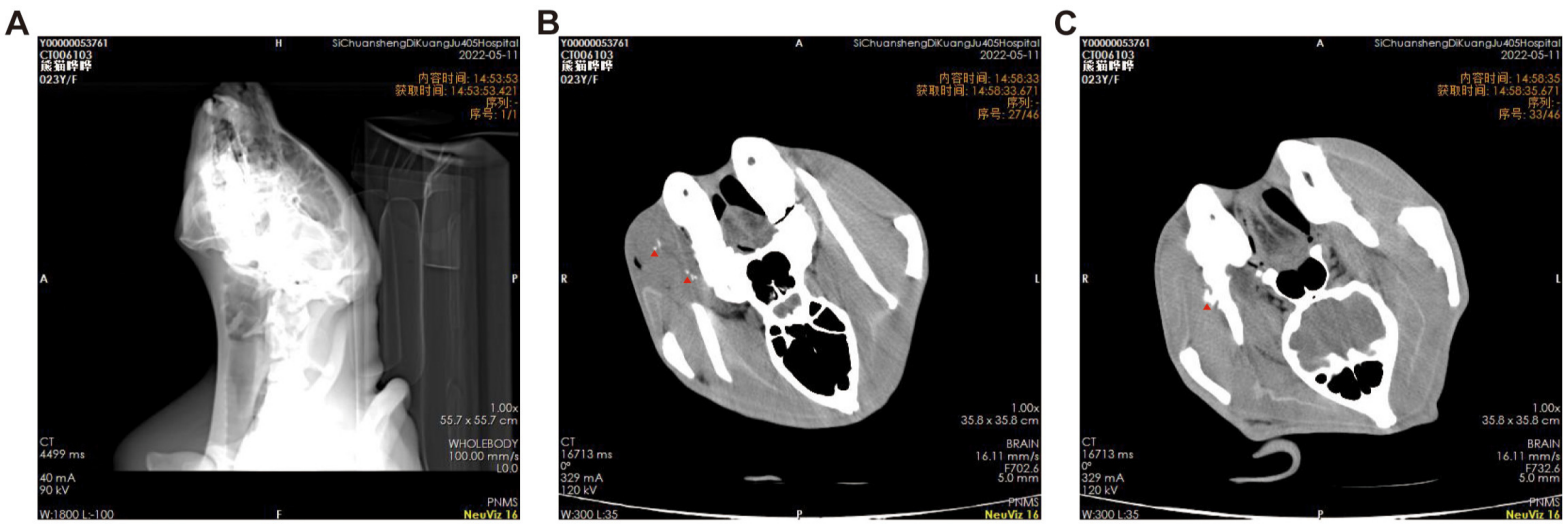
**(A–C)** Cross-sectional computed tomography images of the giant panda's head, the red triangle indicates the location of tumor calcification.

The computed tomography (CT) scan disclosed a soft tissue mass within the right cheek region of the panda, measuring approximately 9.2 cm × 5.0 cm. The mass exhibited uneven density, punctuated by areas of calcification and gas shadows. The demarcation of the lesion was indistinct (Figure 2A), and the cortical bone adjacent to the lesion was hypertrophied and irregular, with significant edema of the surrounding soft tissues (Figure 2B). Sinusitis was also observed (Figure 2C).

### Pathological examination

**Figure 3:**
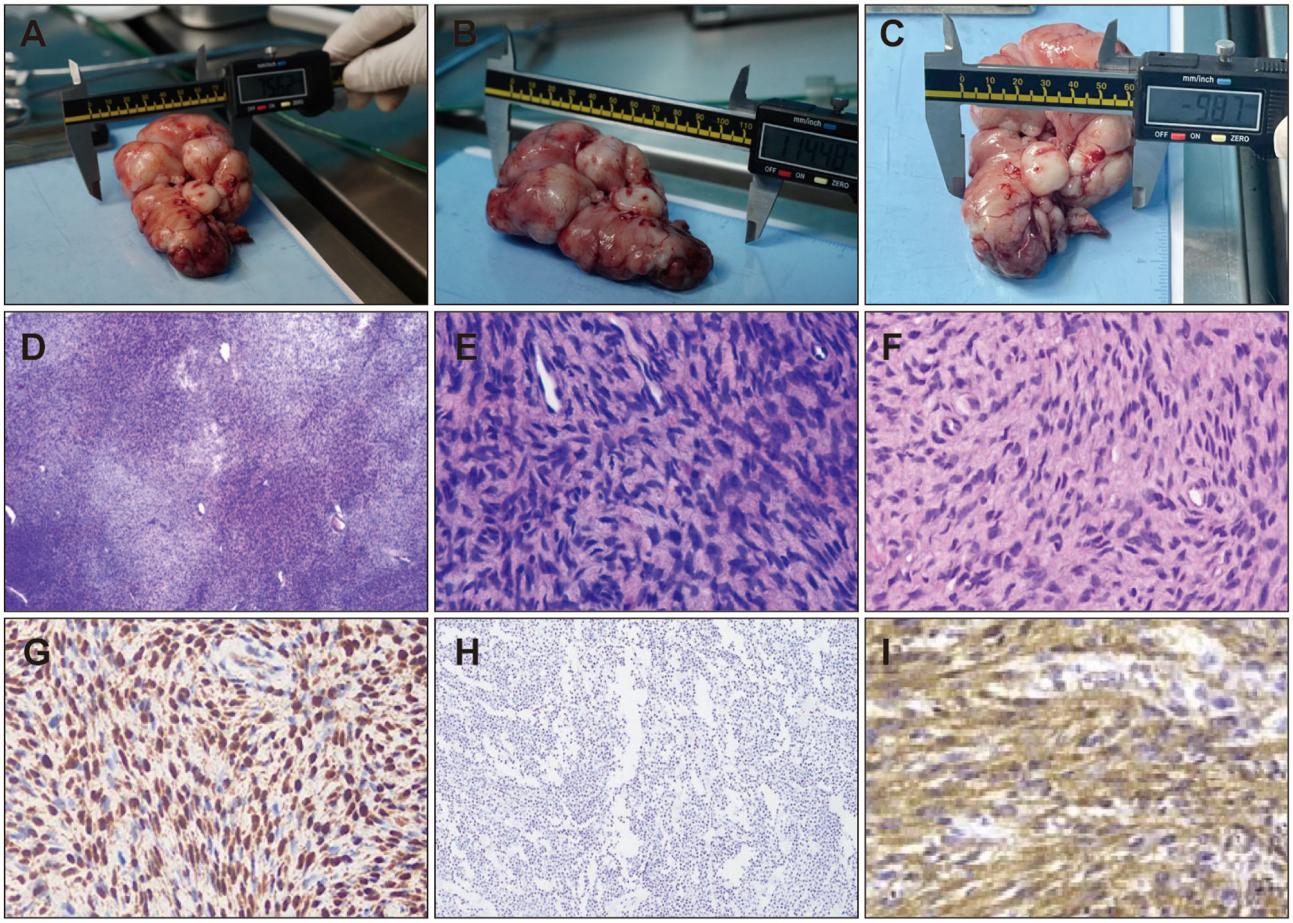
Histopathological Features of Oral Tumor Lesions from Living Giant Pandas. Pathological characteristics of oral tumors collected from living giant pandas **(A–C)**. **(D)** HE staining of tumor tissue (40X), **(E)** HE staining of tumor tissue (100X), **(F)** HE staining of tumor tissue (100X). Immunohistochemical staining of cavitary tumors, **(G)** Positive expression of S100 by immunohistochemistry. **(H)** Positive expression of Vimentin by immunohistochemistry. **(I)** Negative expression of CD34 by immunohistochemistry.

Following the aforementioned diagnostics, a biopsy of the oral mass was performed on the giant panda to obtain tissue samples. The procured samples were photographed, macroscopically observed, measured, and subsequently fixed in PFA solution. Subsequently, histopathological evaluation was conducted via paraffin sectioning, HE staining, and immunohistochemical assays. Macroscopic examination revealed a large tumor, measuring approximately 12 cm × 9 cm × 5 cm. It was situated beneath the buccal mucosa within the oral cavity, appearing as a large, firm plaque with a multinodular, gelatinous surface and rubbery consistency (Figures 3A–C). Histological analysis post paraffin embedding and HE staining revealed that the tumor was composed of numerous neoplastic cells organized in fascicular or storiform arrangements. The neoplastic cells varied in size, exhibiting a spindle or oval morphology, with infrequent mitotic figures, and numerous small blood vessels were noted within the tumor's core (Figures 3D–F). Microscopically, the case demonstrated characteristic features of low-grade malignancy, aligning with a diagnosis of low-grade fibrosarcoma. Further differential diagnosis was conducted using immunohistochemical assays. The immunohistochemical results indicated positive staining for Vimentin (++), focal S100 (+), with negative results for AE1/AE3, EMA, CK7, SMA, CD34, CD31, Desmin, Calponin, CD99, and a low Ki67 labeling index of approximately 1%. Consistent with the immunophenotypic profile of low-grade malignant fibrosarcoma, only Vimentin is expressed in immunohistochemistry; SMA may show positive expression in instances of myofibroblastic differentiation; Desmin, CK, EMA, and CD34 were non-reactive. The immunohistochemical findings corroborate the histopathological diagnosis of low-grade malignant fibrosarcoma (Figures 3G–I).

## Discussion

Due to their unique characteristics, giant pandas can live for decades in artificial breeding environments, resulting in a longer lifespan compared to their wild counterparts. With the extension of the life cycle, the incidence of tumors rises, posing a threat to the lives of geriatric giant pandas. However, the scarcity of early clinical samples hinders effective monitoring of tumor occurrence in geriatric giant pandas. Our report demonstrates that tumor occurrence can be inferred by detecting blood-based tumor markers (PLT and CA724 in this study). Consequently, upon detecting abnormal blood indicators during health assessments, it is imperative to conduct more comprehensive examinations and initiate treatment for tumors at an early stage. This approach can help maintain the population of geriatric giant pandas to a significant extent, while also enriching the clinical data available, thereby aiding in the conservation and growth of the giant panda population.

The typical locations for oral fibrosarcoma in animals include the hard palate and maxillary gingiva (6–8). It presents as a firm, well-circumscribed tumor. The tumor exhibits a nodular, lobulated, or irregular morphology. It is clearly demarcated from the surrounding tissues, and sometimes encapsulated; the consistency is slightly firmer than that of normal tissue; the sectioned surface is pink or off-white, and uniform in appearance (9). Fibrosarcoma tumor cells exhibit size variation, with frequent occurrence of giant cells; the tumor cells display a range of shapes and are markedly pleomorphic; the nuclei of tumor cells are intensely pigmented and frequently exhibit mitotic figures (9); there is a preponderance of tumor cells with a sparse presence of collagen fibers. Diagnosis should be informed by the animal's age, breed, gender, and location characteristics, alongside X-rays and CT scans. However, a definitive diagnosis requires histopathological examination of the excised tissue (9–11). Fibrosarcoma typically shows resistance to radiotherapy and chemotherapy, and is characterized by a high recurrence rate (9, 11, 12). Postoperatively, treatments included injections of cyclophosphamide and cytarabine; however, a recurrence was observed within 14 days (13, 14). Given the unique nature of this case, no additional treatments were administered to the giant panda. The majority of the giant panda's oral bleeding episodes were associated with the consumption of bamboo poles; therefore, the feeding regimen was modified to utilize bamboo shoots in place of poles, aiming to diminish the frequency of bleeding. Following the adjustment, there was a noticeable reduction in the frequency of bleeding. Significant improvements were observed, leading to an enhanced quality of life.

This case report documents the first instance of low-grade oral fibrosarcoma in giant pandas. It has established a standardized and systematic diagnostic protocol, offering valuable data and a reference framework for the diagnosis and prevention of diseases in giant pandas.

## Data Availability

The original contributions presented in the study are included in the article/supplementary material, further inquiries can be directed to the corresponding authors.
